# Dynamic range and optimization strategies for radiochromic film calibration using gradient radiation fields

**DOI:** 10.1002/acm2.14481

**Published:** 2024-08-12

**Authors:** Stevan Pecic, Ivan Belca, Strahinja Stojadinovic, Borko Nidzovic, Milos Vicic, Slobodan Devic

**Affiliations:** ^1^ Faculty of Physics University of Belgrade Belgrade Serbia; ^2^ Department of Radiation Oncology University of Texas Southwestern Medical Center Dallas Texas USA; ^3^ Institute of Oncology and Radiology of Serbia Belgrade Serbia; ^4^ Medical Physics Unit McGill University Montreal Canada; ^5^ Department of Radiation Oncology SMBD Jewish General Hospital Montreal Canada

**Keywords:** dose gradients, dynamic range, optimization, radiochromic film, wedge filter

## Abstract

This investigation aimed to optimize gradient positioning for radiochromic film calibration to facilitate a uniform distribution of calibration points. The study investigated the influence of various parameters on gradient dose profiles generated by a physical wedge, assessing their impact on the field's dose dynamic range, a scalar quantity representing the span of absorbed doses. Numerical parameterization of the physical wedge profile was used to visualize and quantify the impact of field size, depth, and energy on the dynamic range of dose gradients. This concept enabled the optimization of the gradient positioning and estimation of the necessary number of exposures for the desired calibration dose range. An optimization algorithm based on histogram bin height minimization was developed and presented. The maximum dynamic range was achieved with a 20 × 20 ${\mathrm{cm}}^{2}$ field size at 5 cm depth. Optimization of wedge gradient positioning yielded the most uniform dose distribution with 7 exposures for the [1,10] Gy range and 8 exposures for the [1,20] Gy range. Film calibration using gradients centered at 1.6, 3, 3.5, and 7 Gy central axis (CAX), obtained through optimized gradient positioning, was showcased. The presented work demonstrates the potential for an improved film calibration process, with efficient material utilization and enhanced dosimetric accuracy for clinical applications. While the method was described for the use of a physical wedge, the methodology can be easily extended to the use of a more convenient dynamic wedge.

## 1 INTRODUCTION

Contemporary radiation therapy techniques such as IMRT, VMAT, and SBRT employ beam modulations to create steep spatial gradients of absorbed dose. Beyond their well‐established use in treatment planning, dose gradients have garnered interest in radiochromic film dosimetry. Utilizing a gradient field offers the advantage of generating multiple dose values in a single exposure, proving a valuable and efficient approach for calibration purposes in several studies.^1^, ^2^, ^3^ A fundamental example of beam modulation is using a physical wedge to create an intensity gradient across the field. However, the limited span of absorbed dose values produced by a single wedge field hinders its usefulness for calibration. This limitation necessitates combining multiple exposures to create additional calibration points. Ultimately, the goal is to achieve a uniform distribution of calibration points across the entire calibration dose range.

The dose span of a gradient field can be fine‐tuned by adjusting the parameters that influence the shape of the field. In that sense, physical wedges are rudimentary units, having predefined angles. For electronic or dynamic wedges,^4^, ^5^, ^6^ the desired dose distribution is achieved by dynamically moving either one of the collimator jaws or multileaf collimators across the treatment field during delivery. In some machines, a universal wedge is employed; an arbitrary angle smaller than 60 degrees can be produced by combining a 60‐degree physical wedge, housed inside the treatment head, with the open field,^7^, ^8^ effectively reducing the inclination of the dose profile. External parameters that govern the shape of the wedge field are field size, depth of measurement, and beam quality. These parameters were explored in several studies, primarily to determine the wedge factor dependencies.^9^, ^10^, ^11^ Analytical form and modeling of the wedge fields were also explored by Lebron et al.,^12^, ^13^ where the parameterization and numerical segmentation of the wedge profile were demonstrated.

In this study, dose gradients were primarily investigated to understand how the field shape parameters relate to the absorbed dose range. A parameter that estimates the span of the absorbed doses, that is, dynamic range coefficient, was introduced. In particular, the dynamic range of the wedge field is determined by the span of absorbed doses within the normalized wedge profile. Conversely, the dose at the central axis of the profile, multiplied by the dynamic range coefficient yields the range of absorbed doses achievable with the wedge. Additionally, the investigation explored the sensitivity of the dynamic range to measurement conditions. Furthermore, optimizing the wedge gradient positioning was investigated to achieve the desired cumulative dose histogram. The presented formalism may assist in dosimetric applications such as radiochromic film calibration, where multiple wedge fields are combined to optimize the desired calibration range,^3^ thus saving material and time. In general, a similar approach could be used for calibrating other planar dosimetric equipment without introducing new assumptions. Although the presented work pertains to the physical wedge field formalism, it can be generalized to any type of continuous gradient field.

## 2 MATERIALS AND METHODS

### 2.1 Measurement of wedge beam profiles

The wedge dose profiles analyzed in this study were created using an Elekta Versa HD linear accelerator with a 60‐degree physical wedge and 6 MV photon beam. In‐line wedge profiles were obtained using a motorized 3D water phantom equipped with a pair of CC13 ionization chambers (IBA Dosimetry, Schwarzenbruck, Germany). The scanning speed was 1 mm/s with a resolution of 1 mm. The daily output of the linear accelerator before the irradiation was validated utilizing a dedicated ionization chamber (PTW – TN30013) following the IAEA TRS‐398 [13] reference dosimetry protocol. Measurements were carried out for field sizes of {5×5, 10×10, 15×15, 20×20} $cm^{2}$, at depths of {1.6,5,10,20,30} cm, and {6, 10, 15} MV photon beams.

### 2.2 Dynamic range of the wedge field

Recorded wedge profiles were processed to extract the field's slope. First, the numerical gradient was calculated for the profile to determine the position of the physical edge of the field.^13^ Following segmentation, a region between 80% and 20% of the dose profile length was extracted, representing the segment where dose values exhibit a monotonic decrease. As discussed in the previous works,^9^, ^10^, ^11^ the shape of the wedge profile is a function of distance from the source, the size of the field, and the beam quality used. The absorbed dose range of wedge fields can be characterized by a single coefficient called the dynamic range coefficient (DRC). Dose profile slope segmentation facilitates the direct determination of the maximum and minimum dose values relative to the dose at the central axis (CAX). For a wedge field, the DRC is defined as the difference between the maximum and minimum relative doses (RD) within the slope region of the normalized profile.

(1) $$\varepsilon_{W}=RD_{max}-RD_{min}{,|}_{D_{CAX}=1}$$

**FIGURE 1:**
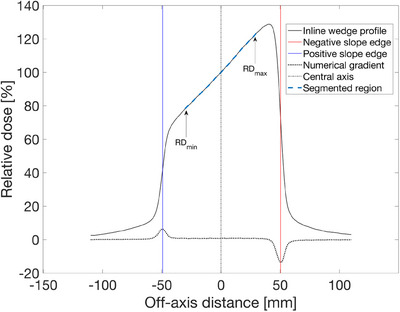
Segmentation of the dose profile slope for a 10 × 10 $cm^{2}$ field produced by a 60‐degree physical wedge. Dashed lines represent numerical gradient values, from which the peaks are identified and used for edge detection.

A graphical representation of wedge slope segmentation and field extraction is presented in Figure 1.

Equation (1) inherently captures the dependence of the DRC on field size. This is because the coefficient is directly proportional to the profile length, which itself increases with a larger field size. The profile shape, on the other hand, is influenced by spectral changes in the beam as it traverses the wedge filter and the depth of measurement. In this work, the reference field was a 10 × 10 $cm^{2}$ wedge field produced by a 6 MV photon beam through the 60‐degree wedge and measured at a depth of 10 cm. The parameters governing the shape of the profile were varied relative to this reference.

### 2.3 Optimization of combining gradients

The key challenge in the study was to identify the minimal number of gradients needed to encompass the entire calibration range while maintaining a uniform distribution (equal density) of measurement points. Gradients centered around lower dose values are denser and thereby produce a greater number of points per unit dose range when the field size is fixed and the resolution of dose measurement is constant. Employing an equal density of calibration points across the entire calibration range enhances the overall robustness of the calibration procedure. This work describes a guideline for achieving equidensity (uniform distribution) of calibration points, considering that only a few exposures are required for this approach. Even if the number of exposures could be assumed by experience, achieving a uniform density of calibration points is nontrivial. This challenge can be considered to be the main aspect of optimizing multigradient usage and the optimization problem can be viewed as minimizing the fluctuation in the resulting dose histogram across all exposures.

This study examined wedge profiles, arguably the most recognized dose gradients in medical dosimetry, due to their simplicity and familiarity. By varying the parameters that influence the shape of the wedge profile, the desired $\varepsilon_{W}$ coefficient can be obtained. In the simplest scenario, for a fixed set of wedge field parameters, the key to achieving a uniform calibration range with multiple gradients primarily involves optimizing the placement of each gradient. In terms of the dynamic range, each gradient position is uniquely identified by its CAX dose, thereby the result of the optimization is given in the form of a series of doses [$D_{1}^{CAX},D_{2}^{CAX},\text{…},D_{N}^{CAX}$], where *N* is the number of exposures.

The concept of dynamic range allows for identifying the position of the gradient boundaries by reinterpreting Equation (1) and calculating the boundaries as:

(2) $$L_{B}=D^{CAX}\left(1-\frac{\varepsilon_{W}}{2}\right)$$

(3) $$U_{B}=D^{CAX}\left(1+\frac{\varepsilon_{W}}{2}\right)$$

The positions of the first and the last gradient, interpreted as gradient boundaries, are trivial and can be determined as:

(4) $$D_{1}^{CAX}=\frac{D_{min}}{1-\frac{\varepsilon_{W}}{2}}$$

(5) $$D_{n}^{CAX}=\frac{D_{max}}{1+\frac{\varepsilon_{W}}{2}}$$

where [$D_{min},D_{max}$] is the deemed dose range. Thus, the problem of finding the optimal gradient arrangement is reduced to positioning the gradients from 2nd to the nth−1, where n is the optimal number of wedge gradients.

### 2.4 Dose histogram equalization

As a result of optimization, multiple‐dose gradients will cumulatively span over the desired calibration range. The overlapping of the gradients leads to an increased density of points in certain parts of the dose range. The position of the gradients is optimized so that the resulting dose histogram is as flat as possible, characterized by bins of equal height. In the context of optimization, this objective is achieved by minimizing the standard deviation of the resultant histogram bin heights.

Let k be the total number of bins in the histogram, and let $x_{i}$ represent the count in the i‐th bin. The standard deviation (σ) is computed using the formula:

(6) $$\sigma =\sqrt{\frac{1}{k}\sum_{i=1}^{k}{\left(x_{i}-\mu \right)}^{2}}$$

where μ represents the mean of the histogram:

(7) $$\mu =\frac{1}{k}\sum_{i=1}^{k}x_{i}$$

The standard deviation of the histogram bins serves as an estimate of the variation in measurement point density across the observed range. This metric is used as the objective function to optimize the selection of dose gradient on the CAX. The resulting set of gradients effectively populates the desired range with calibration points in a uniform manner. To prioritize simplicity, this work adopted a basic optimization approach focused solely on achieving the isodensity of measurement points. The algorithm offers potential for further refinement by introducing a more complex objective function.

### 2.5 Algorithm setup

Optimization was performed using the simulated annealing algorithm^14^ available in the MatlabR2020a optimization toolbox (The MathWorks, USA). Simpler minimization algorithms could justifiably be used in this scenario without compromising generalizability. Nevertheless, this algorithm produced good results within a few seconds, leveraging readily available computing resources and requiring minimal adjustment of default parameters.

The experimental setup described earlier provided DRCs that were subsequently employed to generate synthetic dose gradients for the development of an optimized procedure. The initial guess for the CAX doses from the second to the (N−1)‐th exposure was generated by using a random number generator. The maximum number of iterations was limited to 1000, as further iterations resulted in negligible changes to the objective function value. The optimization temperature function parameter was assigned to an exponential decrease during optimization. Lastly, the temperature was initialized with a value of 1000, influencing the initial trade‐off between exploration and exploitation during the optimization process.

For this numerical experiment, gradients were synthesized with a preset value of 500 measurement points, corresponding to a 10 cm measurement length with a pixel size of 0.2 mm. This setup translates to a resolution of 127 dpi, readily achievable with modern detectors. Bin size was calculated by dividing the dose range by 25 to achieve an adequate number of measurements for data expected to follow a normal distribution, as predicted by the central limit theorem. This approach ensured a well‐defined standard deviation as the objective function. It is important to note the calculated standard deviation is influenced by the chosen histogram bin size. However, if the bin size remains constant across different measurements with varying parameters, it will not affect the optimization outcome.

### 2.6 Experimental validation

The optimization procedure described above was validated through film calibration employing a wedge dose gradient.^3^ Following the optimization procedure detailed in Section 2.3, for the [1,20] Gy calibration range, the proposed algorithm was evaluated by irradiating eight EBT3 films using an Elekta Versa HD linear accelerator with 6 MV photon beams. The beam configuration included a $15\times 15\;cm^{2}$ field size, a 60‐degree physical wedge, and film placement at a depth of 5cm. An equal density of calibration points across the calibration range necessitated 3 single irradiations/films with 1.6, 3.0, and 3.5 Gy CAX, and 5 irradiations/films with 7.0 Gy CAX dose gradients. The five irradiations with 7.0 Gy CAX dose gradients were averaged and processed as a single profile. During irradiation, a control film was included with the irradiated films for the subsequent subtraction of any background signal arising from ambient conditions. After irradiation, the films were scanned using an EPSON 12000XL flatbed scanner, following the protocol by Devic et al.^15^ Scans were saved in 48‐bit color TIFF format, and the green channel was then extracted. Following the method presented by Aldelaijan and Devic,^16^ normalized pixel value (PVnorm) from the green color channel was used as a response function. Upon constructing the PVnorm map, the central region was extracted and 25 central lines were averaged. Lastly, the PVnorm wedge profile was extracted from the resulting line profile, following the numerical gradient edge segmentation shown in Figure 1. From this edge‐segmented profile, a central region encompassing 20% to 80% of the profile length was selected. The resulting selected region was smoothed using the moving mean algorithm with a 25‐pixel window size. For each wedge profile, a corresponding dose profile was generated by multiplying the normalized profile with its corresponding CAX dose. Subsequently, both the original wedge profile and the dose profile underwent segmentation and filtering using the same procedure.

## 3 RESULTS

### 3.1 Profile shape and dose range dependencies

**FIGURE 2:**
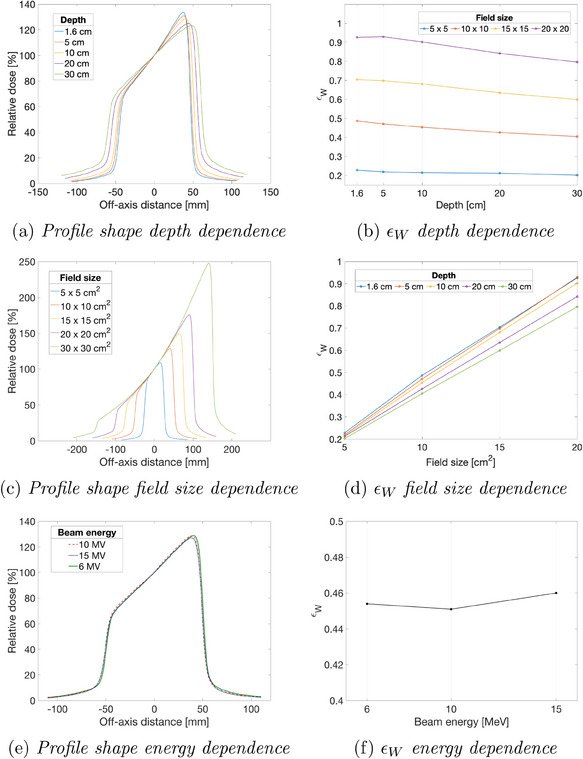
Wedge profile shapes variations and dynamic range coefficient $\varepsilon_{W}$ dependencies.

Figure 2 depicts the influence of varying field size, depth, and beam energy on the acquired wedge profiles alongside the corresponding DRCs. As indicated in Figure 2a, the slope of the wedge profile generally decreases with increasing measurement depth. Beam attenuation with increasing depth causes the photons to lose energy and undergo enhanced scattering in all directions. This phenomenon, coupled with the intrinsic wedge geometry, causes a nonuniform decrease in wedge field profile intensity, with a steeper drop on the heel (thicker) side and a more gradual decrease on the toe (thinner) side. Consequently, the largest dynamic range is achieved at minimal depth (see Figure 2b). As illustrated in Figure 2c, the slope measured at the CAX exhibits no significant variation with increasing field size. However, both the horizontal (run) and vertical (rise) components of the wedge profile increases with field size, effectively creating a longer slope. This extended slope correlates with a linear increase in dynamic range portrayed in Figure 2d. Interestingly, despite the potential influence of beam quality on wedge factors, the profile's shape and dynamic range showed no measurable dependence on beam energy, as evident in Figure 2e,f.

Table 1 presents calculated $\varepsilon_{W}$ for a 6 MV beam with a 60‐degree wedge filter at typical field sizes of $5\times 5\;cm^{2}$, $10\times 10\;cm^{2}$, $15\times 15\;cm^{2}$, and $20\times 20\;cm^{2}$ at depths of 1.6cm, 5cm, 10cm, 20cm, and 30cm. The table indicates that the maximum $\varepsilon_{W}$ is achieved for a $20\times 20\;cm^{2}$ field size at 5cm depth. However, it should be noted that for smaller field sizes this maximum is achieved at 1.6 cm depth. Nevertheless, it is important to note that 1.6 cm is just below the depth of maximum dose ($D_{max}$), and calibrating films around $D_{max}$ is not recommended due to electron contamination.

**TABLE 1:** Dynamic range coefficients $\varepsilon_{W}$ for a 6 MV photon beam with a 60‐degree wedge filter.

Depth/Field size	$5\times 5\;cm^{2}$	$10\times 10\;cm^{2}$	$15\times 15\;cm^{2}$	$20\times 20\;cm^{2}$
1.6 cm	0.228	0.487	0.704	0.926
5 cm	0.219	0.471	0.698	0.929
10 cm	0.215	0.454	0.681	0.902
20 cm	0.212	0.426	0.635	0.842
30 cm	0.202	0.405	0.599	0.796

*Note*: Values are presented for different depths and field sizes.

### 3.2 Optimization results

**FIGURE 3:**
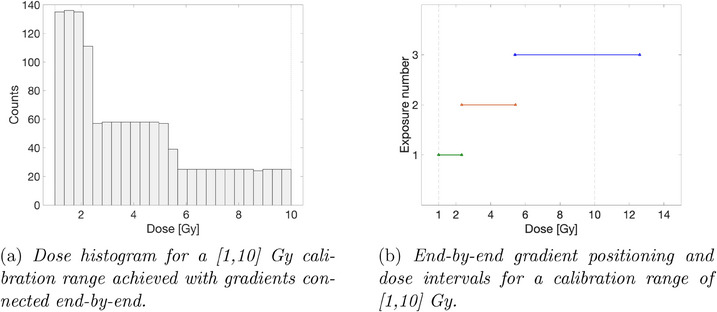
Unoptimized gradient positioning.

The optimization of the gradient positioning was tested for a wedge field with a dynamic range coefficient $\varepsilon_{W}$ of 0.8, for the calibration range [1,10] Gy and 8 exposures. The resulting dose histogram is shown in Figure 3a. The positions of the individual gradients across the observed range and the corresponding dose intervals are illustrated in Figure 3b. When considering dose span criteria, gradients may extend beyond the desired calibration range to guarantee encompassing the entire clinically relevant domain.

**FIGURE 4:**
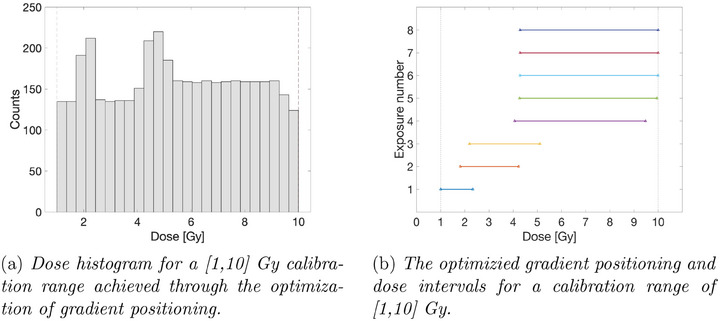
Optimized gradient positioning.

The resulting dose histogram achieved with optimization is shown in Figure 4a. The optimal positions of the individual gradients across the observed range and the corresponding dose intervals are illustrated in Figure 4b. Given that a uniform dose distribution is the goal of optimization, the algorithm preferentially allocates more gradients within the high‐dose region. This accounts for the inherently larger spread of higher dose gradients which leads to a reduced number of data points populating individual histogram bins.

**FIGURE 5:**
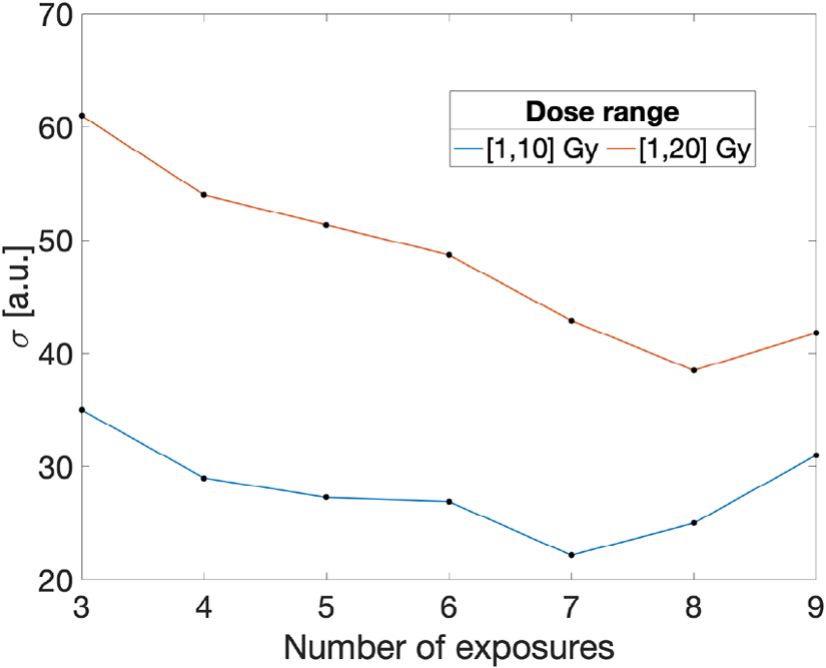
Relationship between the number of exposures and the standard deviation of the dose histogram.

Additionally, Figure 5 represents the relationship between the number of exposures and the standard deviation of the dose histogram. This graph illustrates the impact of exposure count on the uniformity of the dose distribution within the calibration ranges. The presented results demonstrate that the absolute standard deviation reaches a minimum value at 7 exposures in the [1,10] Gy and 8 exposures in the [1,20] Gy range. Equation (A6) (Appendix) provides an estimate of 3 exposures for the [1,10] Gy range and 4 exposures for the [1, 20] range. While it might be tempting to adopt a general rule of thumb of doubling the $N_{range}$ as a reference value, it is important to note that it might not be universally applicable.

### 3.3 Calibration with optimized parameters

**FIGURE 6:**
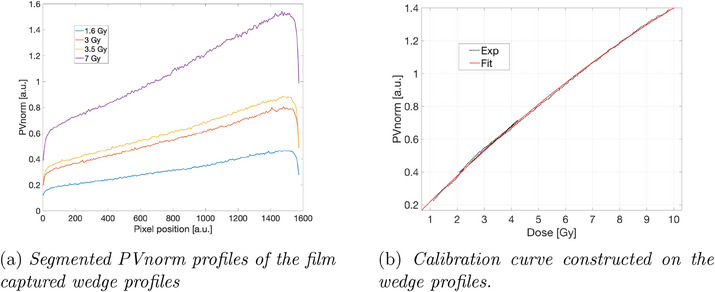
Validation of the optimized film calibration.

Using the optimized parameters, eight films were irradiated with doses of 1.6, 3, 3.5, and 7 Gy CAX, and processed according to the guidelines described in Section 2.6. Raw PVnorm wedge profiles are shown in Figure 6a. The correspondence between the dose and PVnorm profiles allowed for the measured values to be merged into a single series of data points. A second‐order polynomial was fitted to this series, as shown in Figure 6b.

## 4 DISCUSSION

While a typical range of 2 to 4 exposures can span the entire calibration range, achieving a uniform density (isodensity) of dose points often necessitates a higher number of exposures, specifically greater than five as demonstrated in this study. Optimizing the number of exposures has significant implications regarding efficient material use and savings due to the possibility of combining gradients. Compared to homogeneous fields, which typically require 8 to 12 exposures, dose gradients at different dose levels offer a more advantageous alternative. Building on the standard deviation analysis versus exposure count presented earlier, dose gradients at different dose levels necessitate a similar number of exposures compared to homogeneous field calibration. However, the advantage lies in the fact that each gradient can encompass over 500 calibration points. This translates to over 4000 calibration points uniformly distributed across the entire [1,10] Gy dose range. To optimize film utilization, it is recommended to constrain scenarios where the field size is equal to or less than 15×15 $cm^{2}$. This recommendation aligns with the common practice of cutting strips from standard film sheets (typically 8in. × 10in.) and leverages the shorter film dimension (20.32 cm) of EBT3 film for strip creation. As evidenced in Table 1, a 15×15 $cm^{2}$ field size at a depth of 5 cm with a 60‐degree wedge and a 6 MV beam achieves the highest DRC of 0.698.

The presented findings prioritize scenarios where the objective is to achieve a flattened dose histogram. This prioritization is well‐founded when the primary focus lies on characterizing the range and density of acquired dose values. However, specific applications may necessitate a denser distribution of data points within the targeted dose regions, either at lower or higher dose levels. To accommodate such requirements, the optimization framework would require a shift in the objective function. Instead of minimizing standard deviation, the function would transition to minimizing the deviation from a predefined distribution function that incorporates a weighting scheme across the histogram. In the context of radiochromic films, this weighting scheme could be leveraged to model the deviation from an exponentially decaying function, which aligns with the measurement uncertainty distribution reported by Devic et al.^15^ Denser gradients at lower doses enable compensation for film measurement uncertainty by providing a larger number of calibration points in the dose region where the measured signal is low. Interestingly, the unoptimized histogram presented in Figure 3a exhibits a somewhat exponential dependence. However, the introduction of additional overlapping gradients leads to the disappearance of this exponential shape.

For a 10×10 $cm^{2}$ field measured at 10 cm depth, the dynamic range estimation can be derived directly from trigonometric principles, as the angle of the slope is given by definition. In addressing nonmonotonic profiles, a systematic approach involves sorting data points and subsequently applying a consistent analytical template for segmentation. It is noteworthy that gradient‐based optimization techniques traditionally assume a uniform distribution of data points across varying field sizes, reflecting the limitations inherent to detector resolution. However, in more general applications where detector resolution is not a paramount constraint, adjustments to resolution can be implemented. Extending the dynamic range definition can be another strategy to achieve a wider measurable dose distribution. For example, considering data points within a 10% to 90% range, as opposed to the previously employed 20% to 80% interval, would broaden the captured dose variation. In practical applications, however, the parameters influencing profile shape are often constrained to predefined values. This inherent limitation transforms the optimization process into a discrete exploration of the available parameter space.

The findings presented in this work are directly applicable to dynamic wedges, given the increasing adoption of advanced beam modulation technologies that supersede traditional physical wedges. Furthermore, these conclusions have the potential to significantly enhance the efficacy and precision of other proposed calibration procedures that leverage dose gradients for film calibration.^1^, ^2^, ^3^ Future research endeavors may focus on developing more specialized optimization algorithms for gradient positioning within wedge fields. These algorithms could address the challenges of nonmonotonic profiles and accommodate variable detector resolution requirements. Additionally, exploring the application of optimized gradient positioning to other dosimetric equipment has the potential to broaden the work's utility within radiation therapy quality assurance programs.

## 5 CONCLUSIONS

This investigation employed a comprehensive analysis of dose gradients generated by a motorized 60‐degree physical wedge. The primary objective was to elucidate the interplay between these gradients and various parameters governing profile shape and attainable dose range. These insights provide valuable considerations for optimizing gradient positioning for wedge fields, with the potential to significantly improve film calibration methodologies. Optimized gradient positioning in this study demonstrated the capability to achieve uniform dose distributions with a reduced number of exposures compared to conventional homogeneous calibration techniques. These findings hold significant implications for material conservation and present a robust alternative for film calibration procedures that traditionally rely on homogeneous fields.

## AUTHOR CONTRIBUTIONS

All authors contributed to the conception, design, analysis, and critical revision of the manuscript.

## CONFLICT OF INTEREST STATEMENT

The authors declare no conflicts of interest.

## CALIBRATION DOSE RANGE CRITERION

Using Equations (2)–(5), it is possible to estimate the number of exposures necessary to span over the desired calibration range, given the dynamic range coefficient $\varepsilon_{W}$. Here is the rationale: assuming there is no overlap between the gradients, the number of exposures necessary to span over the calibration range is identical to the number of exposures when the gradients are connected by ends. From Equation (3) the lower boundary of the second gradient is given as:

(A1)
$$D_{2}^{LB}=D_{1}^{CAX}\left(1+\frac{\varepsilon_{W}}{2}\right)=D_{min}\left(\frac{1+\frac{\varepsilon_{W}}{2}}{1-\frac{\varepsilon_{W}}{2}}\right)$$

(A2)
$$D_{2}^{CAX}=\frac{D_{min}^{LB}}{1-\frac{\varepsilon_{W}}{2}}=D_{1}^{CAX}\left(\frac{1+\frac{\varepsilon_{W}}{2}}{1-\frac{\varepsilon_{W}}{2}}\right)=D_{min}\frac{\left(1+\frac{\varepsilon_{W}}{2}\right)}{{\left(1-\frac{\varepsilon_{W}}{2}\right)}^{2}}$$

It can be shown by induction that the CAX dose of the *N*‐th gradient can be written as:

(A3)
$$D_{N}^{CAX}=D_{min}\frac{{\left(1+\frac{\varepsilon_{W}}{2}\right)}^{N-1}}{{\left(1-\frac{\varepsilon_{W}}{2}\right)}^{N}}$$

whose upper boundary is given by Equation (3):

(A4)
$$D_{N}^{UB}=D_{min}{\left(\frac{1+\frac{\varepsilon_{W}}{2}}{1-\frac{\varepsilon_{W}}{2}}\right)}^{N}$$

The criterion for achieving the [$D_{min},D_{max}$] dose range is that the upper boundary of the *N*‐th (last) gradient exceeds the upper limit of the needed dose range:

(A5)
$$D_{N}^{UB}>D_{max}$$

Combined with expression A4, this criterion can be writen as:

(A6)
$$D_{min}{\left(\frac{1+\frac{\varepsilon_{W}}{2}}{1-\frac{\varepsilon_{W}}{2}}\right)}^{N}>D_{max}$$

By taking the logarithm of Equation (A6) and rearranging the resulting equation, the minimum number of exposures to encompass the entire calibration range is given as:

(A7)
$$N_{range}>\frac{\log\frac{D_{max}}{D_{min}}}{\log\frac{1+\frac{\varepsilon_{W}}{2}}{1-\frac{\varepsilon_{W}}{2}}}$$
